# Surgical Observations

**Published:** 1877-11

**Authors:** 


*Surgical Observations, with cases and operations.* By J. Mason Warren, M. D., Surgeon to the Massachusetts General Hospital; Follow of the American Academy of Arts and Sciences, etc. Boston: Ticknor & Fields, 1867. For sale by A. Williams & Co., Boston.

This excellent work has been kept in store for the last ten years, but is now placed before the public at a greatly reduced price. The old price was $10,00, now it can be had for $3,50. Three hundred and seventy-three cases are presented, which are illustrated by fourteen wood cuts and five plates. Many of the cases presented are rare, while they are all reported in a masterly and scholarly manner. We have not the space to notice the cases individually, but we must say that most of them will repay close attention. The price is so low that every one should secure it. C.

